# Prognostic significance of CD44 in human colon cancer and gastric cancer: Evidence from bioinformatic analyses

**DOI:** 10.18632/oncotarget.9998

**Published:** 2016-06-14

**Authors:** Pu Xia, Xiao-Yan Xu

**Affiliations:** ^1^ Department of Cell Biology, College of Basic Medical Science, Liaoning Medical University, Jinzhou, Liaoning, P.R. China; ^2^ Department of Pathophysiology, College of Basic Medical Science, China Medical University, Shenyang, Liaoning, P.R. China

**Keywords:** CD44, The Cancer Genome Atlas, gastric cancer, colon cancer, bioinformatic analyses

## Abstract

CD44 is a well-recognized stem cell biomarker expressed in colon and gastric cancer. In order to identify whether CD44 mRNA could be used as a prognostic marker in colon and gastric cancer, bioinformatic analyses were used in this study. cBioPortal analysis and COSMIC analysis were used to explore the CD44 mutation. CD44 mRNA levels were evaluated by using SAGE Genie tools and Oncomine analysis. Kaplan-Meier Plotter was performed to identify the prognostic roles of CD44 mRNA in these two cancers. In this study, first, we found that low alteration frequency of CD44 mRNA in colon and gastric cancer. Second, the high CD44 mRNA level was found in colon and gastric cancer, and it correlated with a benign survival rate in gastric cancer. Third, CD4 and CD74 may be used as markers to predict the prognosis of colon and gastric cancer. However, the deep mechanism(s) of these results remains unclear, further studies have to be performed in the future.

## INTRODUCTION

Cancer has become a major health problem and a large economic burden in China [1]. According to the data of The National Central Cancer Registry of China (NCCR), gastric cancer and colorectal cancer are two commonly diagnosed cancers in both man and women in china [1].

**Figure 1:**
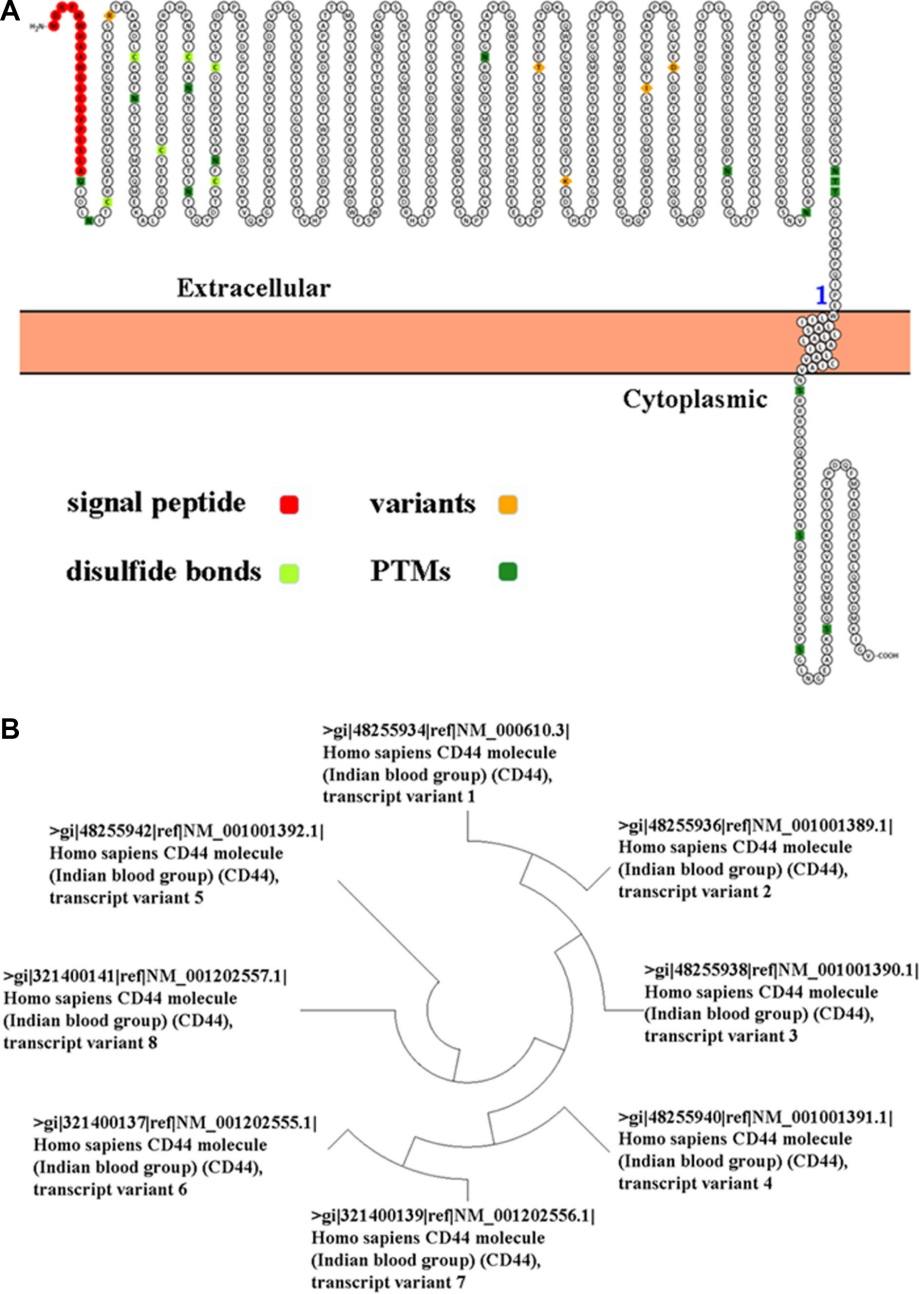
(**A**) Ideograph of CD44 protein was generated by using Peptide Atlas. (**B**) An evolutionary tree of CD44 transcript variant was generated by the maximum likelihood analysis.

In recent studies, cancer stem cell (CSC) hypothesis has been proposed to explain the pathogenesis of cancers [2]. To understand the exact biological features of CSC, the key thing is to isolate CSC from cancer cells. A series of new studies indicated that CD44 expression was elevated in cancer stem-like cells in many kinds of cancer [3]. The human CD44 gene is located on chromosome 11p13 [4]. CD44 protein was initially identified as the receptor for hyaluronic acid (HA), and involved in proliferation, differentiation and motility of both normal and cancer cells [5]. CD44 protein contains an N-terminal HA-binding link-homology module, stem region, transmembrane domain, and short C-terminal cytoplasmic domain (Figure 1A) [6]. CD44 isoforms are generated by alternative splicing of 10 variant exons (v1-v10) in the extracellular domain [7]. Molecular weight of CD44 isoforms ranges from 85 kDa (CD44s, standard version) to 250 kDa (CD44 v3-v10) (Supplementary Figure S1) [8].

Following the identification of various CD44 transcripts (Figure 1B and Supplementary Table S1), the studies for CD44 became complex. For example, CD44s suppresses metastasis, but CD44v7-10 facilitates invasion in prostate cancer [9]. CD44 expression in the colon cancer tissue was substantially higher than that in normal mucosa and it correlates with cancer progression and aggressiveness [10]. CD44 expression was positively correlated with lymph node and distant metastasis, and poorer outcome of gastric cancer patients [11]. In this study, we evaluated the significance of CD44 mRNA in human colon and gastric cancer by using The Cancer Genome Atlas (TCGA) data portal.

## RESULTS

### CD44 mutation in colon and gastric cancers

**Figure 2:**
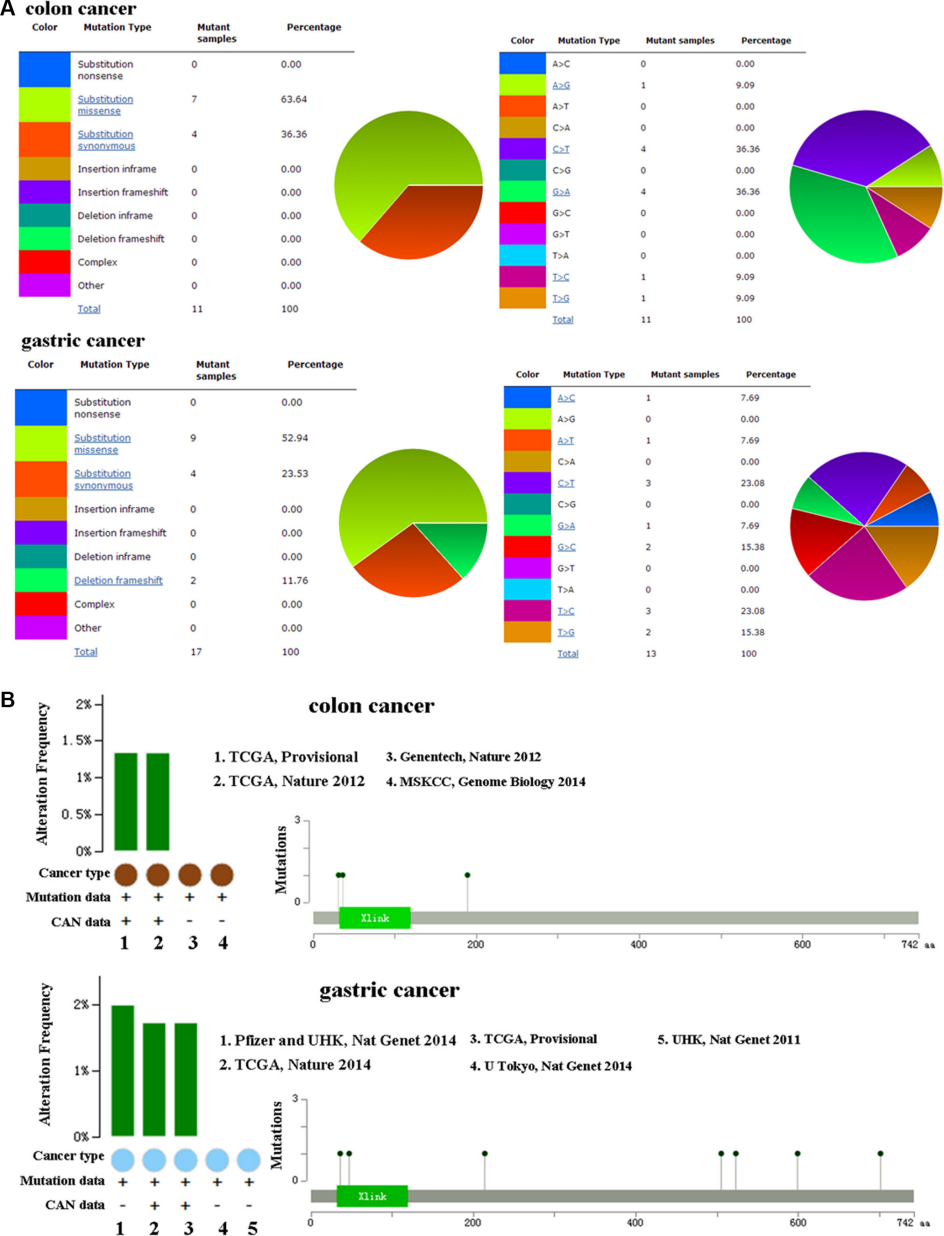
(**A**) Pie-chart showed the percentage of the mutation type of CD44 in colon and gastric cancer according to COSMIC database. (**B**) Alteration frequency of CD44 mutation in colon and gastric cancer was analyzed by using BioPortal.

The pie chart that included the information of mutations of substitution missense, nonsense, synonymous, and insertion frame shift was generated by using COSMIC. Substitution missense rate is 63.64% and substitution synonymous rate is 36.36% of mutant samples of colon cancer (Figure 2A). Mutant samples of gastric cancer have 52.94% substitution missense, 23.53% substitution synonymous, and 11.76% deletion frameshift (Figure 2A). Colon cancer has 36.36% C > T and 36.36% G > A mutation in CD44 coding strand, and 23.08% C > T and 23.08% T > C mutation in gastric cancer (Figure 2A). Alteration frequency of CD44 mutation in colon and gastric cancer was analyzed by using BioPortal. Less than 2% mutation in the patients with colon and gastric cancer was observed (Figure 2B).

### CD44 mRNA in colon and gastric cancer tissues

**Figure 3:**
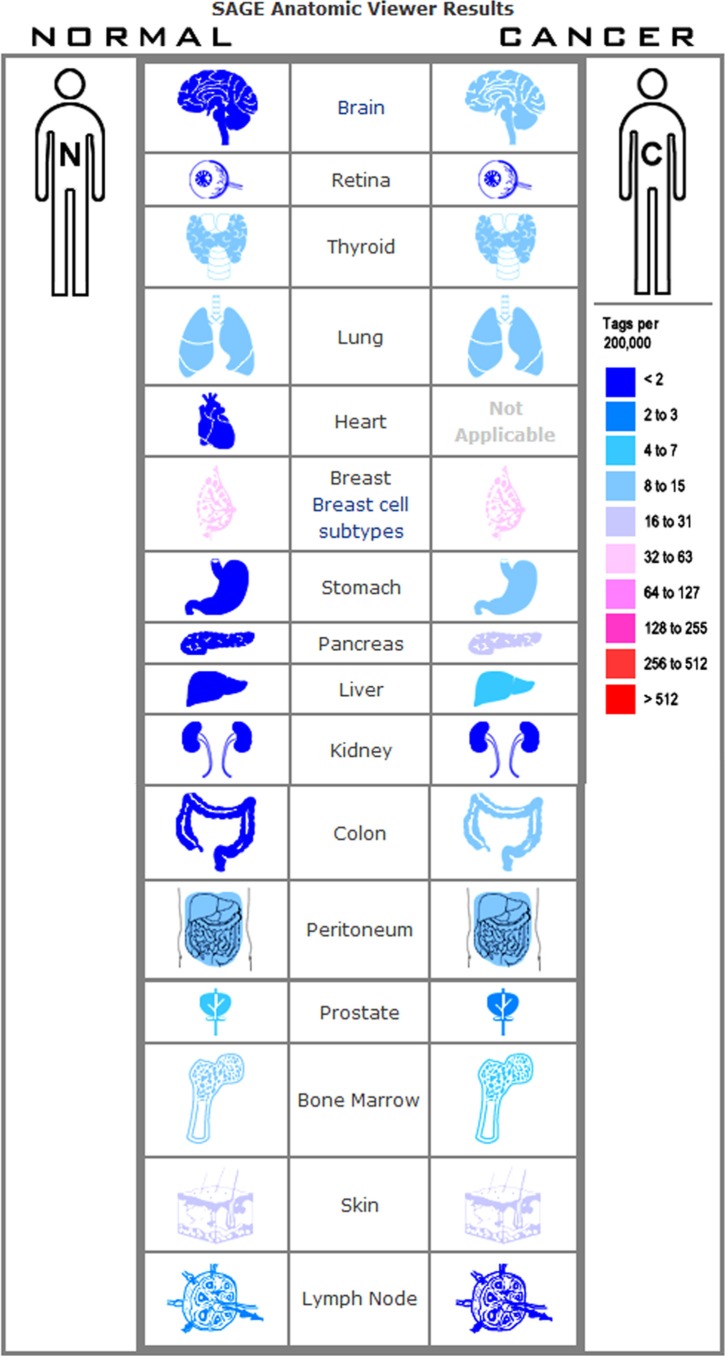
Expression profile for CD44 in human cancers found by the SAGE DGED

**Figure 4:**
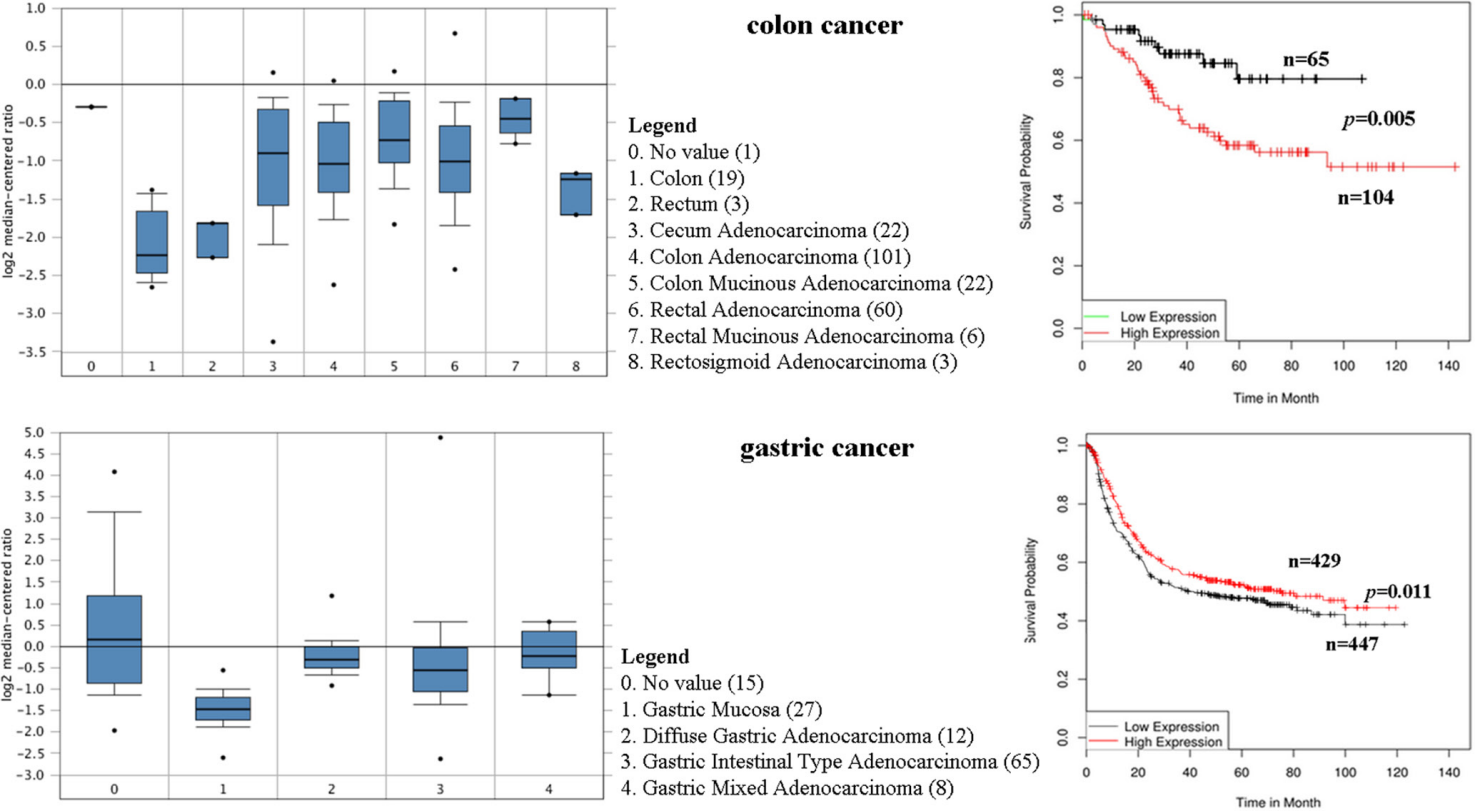
CD44 mRNA was evaluated in colon and gastric cancer by using Oncomine analysis Prognostic significance of CD44 enriched in colon and gastric cancer.

The expression profile of CD44 was found by using the SAGE Digital Gene Expression Display. Higher levels of CD44 mRNA are mainly in brain, stomach, pancreas, liver, and colon cancer tissues, compared with their matched normal tissues (Figure 3). Oncomine analysis of cancer vs. normal tissue showed that CD44 mRNA was significantly higher in colon adenocarcinoma, colon mucinous adenocarcinoma, diffuse gastric adenocarcinoma, gastric intestinal type adenocarcinoma, gastric mixed adenocarcinoma (Figure 4). Kaplan-Meier analysis revealed that high CD44 mRNA was correlated with a benign survival rate in gastric cancer (*P* = 0.011), while the opposite role of CD44 mRNA was observed in colon cancer (*P* = 0.005) (Figure 4). In addition, we analyzed the prognostic roles of CD44 mRNA in subtypes of gastric cancer. High level of CD44 mRNA could improve the survival rate in the patients with intestinal-type gastric cancer (*P* = 0.035) (Supplementary Figure S2). Just the opposite role was observed in the patients with diffuse-type gastric cancer (*P* = 0.0064) (Supplementary Figure S2). No influence of CD44 mRNA on mixed-type gastric cancer was found (*P* = 0.14, Supplementary Figure S2). Opposite roles of CD44 mRNA were also found in well-differentiated gastric cancer and poorly/moderately differentiated gastric cancer (Supplementary Figure S2).

### Coexpression of CD44 mRNA

**Figure 5:**
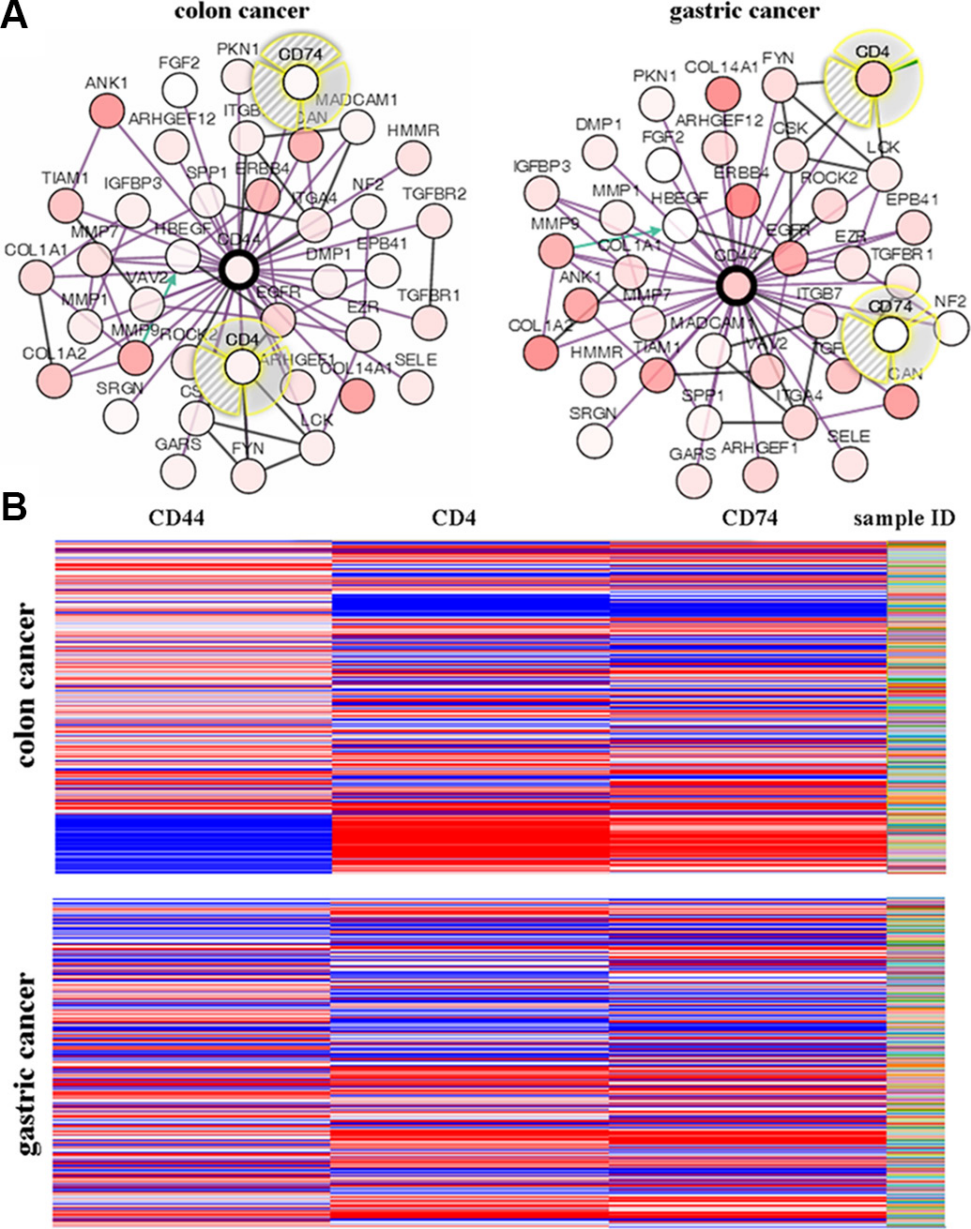
(**A**) Interaction genes of CD44 were analyzed by using Oncomine. (**B**) Relationships of CD44, CD4 and CD74 in colon and gastric cancer were analyzed by using the UCSC Cancer Genomics Browser.

Coexpression genes of CD44 were shown in Figure 5A. Among these genes, we focused on CD4 and CD74. Figure 5B illustrated the whole view for CD44, CD4, and CD74 mRNA of colon and gastric cancer samples based on TCGA database. The heatmaps of CD44 mRNA in colon cancer were strikingly opposite to CD4 and CD74 (Figure 5B). However, no obvious trend was found among CD44, CD4, and CD74 mRNA in gastric cancer (Figure 5B).

## DISCUSSION

Many studies have shown the association of CD44 polymorphisms with cancer risk prediction and prognosis [12–16]. CD44 rs187115 was associated with an increased risk of tumor-related death and lower drug sensitivity in sarcoma [13]. CD44 rs187115 is also correlated with bone metastasis and tumor stage in non small cell lung cancer (NSCLC) patients [14]. Although CD44 polymorphism is very important for cancers, few reports showed the roles of CD44 polymorphism in colon and gastric cancer. CD44 rs8193 is an independent prognostic marker for high-risk stage II and stage III colon cancer patients [15]. CD44 rs187116 could predict disease recurrence in gastric cancer patients, and the single nucleotide polymorphism (SNP) was associated with CD44 isoform switching [16]. In this study, based on the results of bioinformatic analyses, we speculated there may be two reasons for a few studies on CD44 polymorphism in colon and gastric cancer. One reason is that the major proportion of mutation in these two cancers is synonymous mutations. Another reason is that low alteration frequency was observed in colon and gastric cancer.

In our study, higher CD44 mRNA was identified in both colon and gastric cancer by using TCGA database. This finding was consistent with previous studies. Jing et al. [17] found that CD44 mRNA was increased in colorectal cancer tissues than that in matched normal tissues. Wang et al. [18] performed a quantitative review and confirmed higher CD44 levels in gastric cancer. Furthermore, we found that CD44 mRNA is associated with poor overall survival (OS) in colon cancer, while with begin OS in gastric cancer. However, the prognostic roles of CD44 protein in colon and gastric cancer remain controversial. Both Lugli et al. [19] and Hong et al. [20] found that loss of membranous CD44 was linked to the worse survival in colorectal cancer. In the Pitule's study, no relation was found between CD44 expression and OS of colorectal cancer patients [21]. Huh et al. [22] found that CD44 overexpression is an independent unfavorable prognostic factor for OS in colorectal cancer. Similar controversies also existed in gastric cancer [23–25]. The reason for these controversies is very complex. First of all, the heterogeneity of CD44 protein varies in different cell types and growth conditions [7, 8]. So, the protein sequence with immunogen is different in each cancer cell (Supplementary Figure S1). Second, it is difficult to make a definite conclusion for the limited sample size of a single study. Based on the reasons above, it seems that CD44 mRNA used as a prognostic marker is better than CD44 protein.

Another finding in this study is that CD4 and CD74 may be used as markers to predict the prognosis of colon and gastric cancer, but not the markers for cancer stem cell. CD4^+^ T cell-dependent anti-tumor immunity is the immunological driver behind beneficial clinical responses to cancer [26]. In this study, we confirmed CD44 was strikingly opposite to CD4 in colon cancer. It indicated that CD44 expression may regulate anti-tumor immunity by suppressing CD4 expression. CD74, a transmembrane glycoprotein, associates with the major histocompatibility complex (MHC) Class II and plays important roles in the intratumoral immune response [27–29]. CD74 was overexpressed in thyroid malignancy, malignant pleural mesothelioma, and head and neck squamous cell carcinomas, and it is associated with advanced tumor stage [30–32]. Shakib et al. [33] found that both CD44 and CD74 proteins were significantly overexpressed in oral squamous cell carcinoma (OSCC) patients. These results indicated that CD4 and CD74 were not expressed in CD44^+^ cancer stem-like cells, and their expression in immune cells is regulated by CD44 induced-antitumor immune response. However, further studies are required to confirm the roles of CD4 and CD74 in cancers.

There are three main conclusions in this study: 1) Low alteration frequency was observed in colon and gastric cancer; 2) High CD44 mRNA was found in colon and gastric cancer than that in matched normal tissues, and it correlated with a benign survival rate in gastric cancer; 3) CD4 and CD74 may be used as markers to predict the prognosis of colon and gastric cancer, but not the markers for cancer stem cell. However, further studies have to be performed in the future.

## MATERIALS AND METHODS

### Phylogenetic analysis

Maximum likelihood (ML), neighbor-joining (NJ), and Bayesian Markov chain Monte Carlo (MCMC) approaches were used to determine the phylogenetic relationships of CD44 transcript variant 1–8. Evolutionary tree was generated by using the MEGA 5.1 software.

### COSMIC analysis for CD44 mutations

The Catalog of Somatic Mutations in Cancer (COSMIC) database (http://www.sanger.ac.uk/cosmic/) was used for analysis CD44 mutations. Pie charts were generated for a distribution overview and substitutions on the coding strand in colon cancer and gastric cancer.

### cBioPortal analysis for alteration frequency of CD44

Alteration frequency of CD44 mRNA in colorectal adenocarcinoma and stomach adenocarcinoma was performed using BioPortal for Cancer Genomics (http://www.cbioportal.org). All searches were performed according to the cBioPortal's online instructions. The database query was based on mutation and altered expression of the CD44 in colorectal adenocarcinoma (TCGA, Provisional; TCGA, Nature 2012; Genentech, Nature 2012; MSKCC, Genome Biology 2014) and stomach adenocarcinoma (Pfizer and UHK, Nat Genet 2014; TCGA, Nature 2014 TCGA, Provisional; U Tokyo, Nat Genet 2014; UHK, Nat Genet 2011).

### Serial analysis of gene expression (SAGE)

All available published SAGE data were used for analysis of CD44 gene expression in normal and cancerous tissues. Digital CD44 gene expression profiles were analyzed using SAGE Genie tools (http://www.ncbi.nlm.nih.gov/SAGE/).

### Oncomine database analysis

CD44 mRNA levels in colon and gastric cancer tissues were compared with their matched normal tissues by using TCGA datasets in Oncomine database (http://www.oncomine.org). The threshold used to obtain the most significant probes of the queried gene for each microarray data included a two-fold difference in expression between cancers and normal tissues with a *P*-value < 1 × 10^−4^. Genes coexpressed with CD44 were also analyzed and the map was generated by using Cytoscape 3.3.0.

### Kaplan-Meier plotter analysis

The prognostic value of CD44 gene in colon cancer and gastric cancer was analyzed using Kaplan-Meier Plotter (http://kmplot.com/analysis/) and PPISURV (http://www.bioprofiling.de). Overall survival of the patient with high and low levels of CD44 was shown by using a Kaplan-Meier survival plot.

### Genomic data and views

The UCSC Cancer Genomics Browser (https://genome-cancer.ucsc.edu/) was used for integrative visualization of large, complex genomic datasets. According to the cluster results of Oncomine database analysis, we entered CD44, CD4, and CD74 into the search box to perform a search by using Genes viewing mode.

## SUPPLEMENTARY MATERIALS FIGURES AND TABLE


