# Supplementary material for: Prognostic significance of CD44 in human colon cancer and gastric cancer: Evidence from bioinformatic analyses
Source: Oncotarget. 2016 Jun 14;7(29):45538–46. doi: 10.18632/oncotarget.9998 (PMC5216740; doi:10.18632/oncotarget.9998)
Supplement: Supplementary file 1 [file oncotarget-07-45538-s001.pdf]

Prognostic significance of CD44 in human colon cancer and gastric cancer: Evidence from bioinformatic analyses

Supplementary Materials

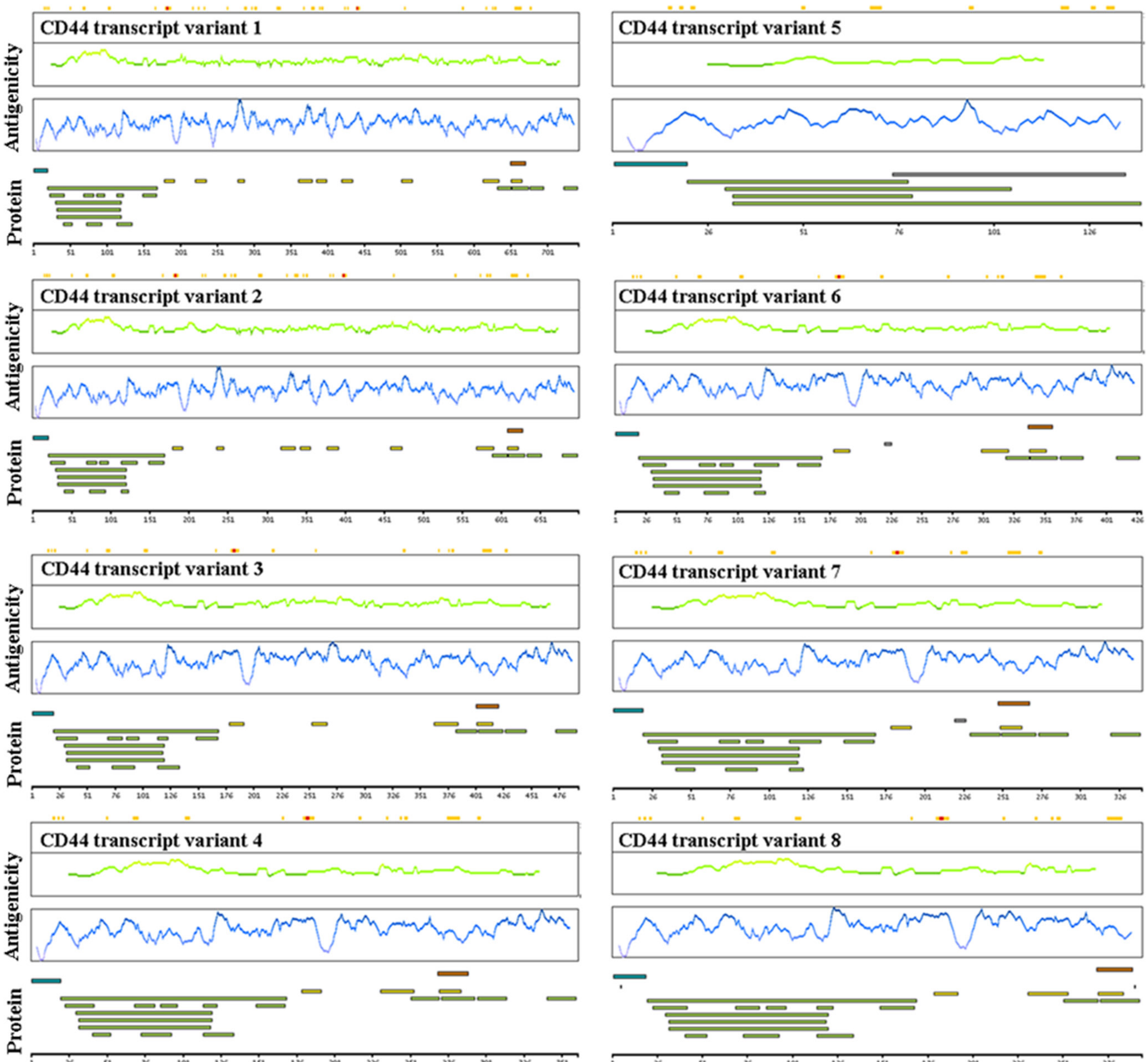

Supplementary Figure S1: Ideograph of antigenicity of CD44v1-8 was generated by using Human Protein Atlas.

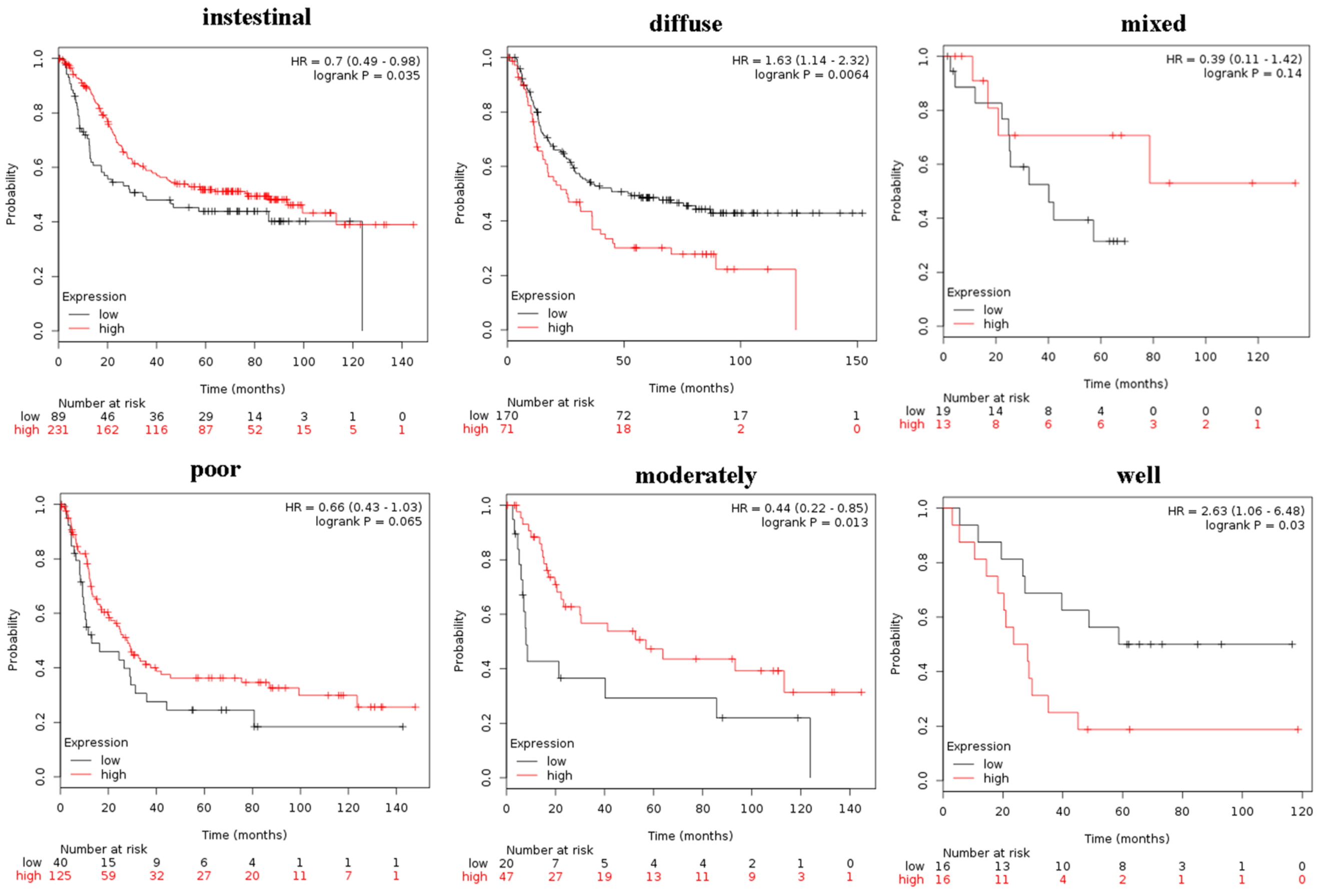

Supplementary Figure S2: Univariate Kaplan-Meier survival analysis of CD44 in subtypes of gastric cancer was performed using datasets compiled at KM-plotter. intestinal: intestinal-type gastric cancer; diffuse: diffuse-type gastric cancer; mixed: mixed-type gastric cancer; well: well differentiated gastric cancer; poor: poorly differentiated gastric cancer; moderately: moderately differentiated gastric cancer.

Supplementary Table S1: Estimates of evolutionary divergence between sequences

	[1]	[2]	[3]	[4]	[5]	[6]	[7]	[8]
[1]								
[2]	0.000							
[3]	0.000	0.000						
[4]	0.000	0.000	0.000					
[5]	0.446	0.446	0.446	0.446				
[6]	0.000	0.000	0.000	0.000	0.446			
[7]	0.000	0.000	0.000	0.000	0.446	0.000		
[8]	0.000	0.000	0.000	0.000	0.446	0.000	0.000	

[1] #gi|48255935|ref|NP_000601.3|_CD44_antigen_isoform_1_precursor_Homo_sapiens.
[2] #gi|48255937|ref|NP_001001389.1|_CD44_antigen_isoform_2_precursor_Homo_sapiens.
[3] #gi|48255939|ref|NP_001001390.1|_CD44_antigen_isoform_3_precursor_Homo_sapiens.
[4] #gi|48255941|ref|NP_001001391.1|_CD44_antigen_isoform_4_precursor_Homo_sapiens.
[5] #gi|48255943|ref|NP_001001392.1|_CD44_antigen_isoform_5_precursor_Homo_sapiens.
[6] #gi|321400140|ref|NP_001189485.1|_CD44_antigen_isoform_7_precursor_Homo_sapiens.
[7] #gi|321400142|ref|NP_001189486.1|_CD44_antigen_isoform_8_precursor_Homo_sapiens.
[8] #gi|321400138|ref|NP_001189484.1|_CD44_antigen_isoform_6_precursor_Homo_sapiens.
